# Ultrasound image guided injection of botulinum toxin for the management of spasticity: A Delphi study to develop recommendations for a scope of practice, competency, and governance framework

**DOI:** 10.1016/j.arrct.2023.100299

**Published:** 2023-09-29

**Authors:** Stephen A. Ashford, Gary Morris, Michael J. Smith

**Affiliations:** a Regional Hyper-acute Rehabilitation Unit, London North West University healthcare NHS Trust & Department of Palliative Care, King's College London, London, England; b School of Healthcare Sciences, Cardiff University, Cardiff, Wales; c Hywel Dda University Health Board, Carmarthen, Wales

**Keywords:** Botulinum Neurotoxins, Delphi method Muscle spasticity, Rehabilitation, Ultrasound Interventional

## Abstract

**Objective:**

To establish a scope of practice, competency (through education) and governance framework for ultrasound image guided injection of botulinum toxin in the management of spasticity

**Design:**

Delphi study

**Setting:**

International, web-based survey

**Participants:**

A purposively selected multidisciplinary (physicians, physiotherapists, occupational therapists) panel of experts (n=15) in the use of ultrasound image guided injection of botulinum toxin for management of spasticity. Panel members were predominantly based in the UK (11/15).

**Interventions:**

In round 1, open-ended questions were posed relating to potential scope of practice for ‘ultrasound imaging in spasticity management’; (specifically relating to ultrasound image guided injection of Botulinum Toxin) education/competency and governance considerations. In round 2, respondents were asked to rate their level of agreement with the statements generated.

**Outcome measures:**

5-point Likert scale used for rating the statements. Threshold for consensus agreement was set at 70% or above.

**Results:**

Three different scopes of practice relating to ultrasound imaging in spasticity management were accepted. The primary scope of practice was the use of ultrasound imaging to guide safe and accurate delivery of botulinum toxin. Relating to this primary scope, 7 competency requirements were agreed relating to areas including image optimization and interpretation, needle visualization and safety. A singular, broad governance statement was generated.

**Conclusion:**

Relating specifically to guided injection of botulinum toxin for management of spasticity, we present a scope of practice, competency, and governance framework. These are integrated within a framework approach to provide a mechanism for increased patient access to accurate, safe, and effective focal spasticity treatment. The framework supports focused training routes, greater inter-profession communication and wider clinical community engagement in spasticity management using this modality.

## Graphical Abstract

**Figure:**
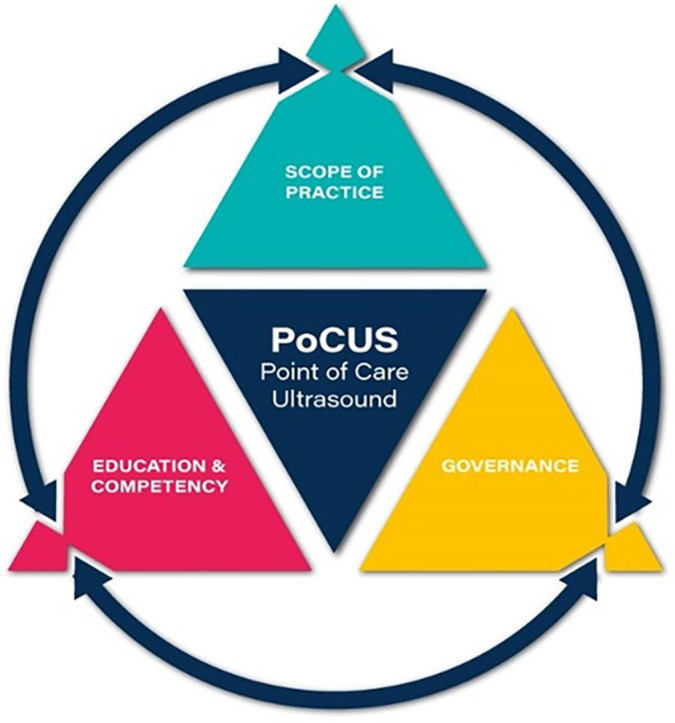

Spasticity is a marked clinical problem in rehabilitation environments and in long-term management of people after central nervous system injuries and conditions. Spasticity occurs in approximately one-third of people post-stroke and in up to 75% of people with severe traumatic brain injury.^1^^,^^2^ Spasticity is defined by the EU-SPASM group as ‘a disorder of sensory-motor control resulting from an upper motor neurone lesion, presenting as intermittent or sustained involuntary activation of muscles’.^3^ If left un-treated, spasticity can result in a vicious cycle of clinical deterioration, in which unopposed contraction (spastic dystonia) in affected muscle groups can lead to joint deformities, skin breakdown (pressure injury), further functional impairment and pain.^4^, ^5^, ^6^

Botulinum toxin (BoNT) is a core treatment applied widely in clinical practice for focal spasticity.^7^ BoNT is injected into the specific muscles where spasticity is identified and causing activity limitation in terms of function or personal care.^8^ Partial or complete delivery into muscles that are not the therapeutic target can result in partial or complete absence of the intended therapeutic effect; and may lead to impaired activity in muscles that may be relied upon for function.^6^ Though less commonly administered, phenol is also used for focal spasticity management. It has the advantage of being longer acting and can be used either to target motor endpoints of the muscle or motor nerve trunks,^7^^,^^9^ causing weakness and minimizing the spasticity.

Accuracy of delivery for BoNT injection in spasticity management is important for 2 main reasons: (1) to ensure optimal therapeutic benefit; and (2) to reduce the risk of iatrogenic harm (unintended consequences of medical intervention).^8^ The delivery of BoNT into non-target tissue may conceivably cause harm, including where it enters the bloodstream. In parallel, a particular risk associated with venipuncture in spasticity management is that many patients may be anticoagulated for stroke prevention purposes, which results in a small but present increased risk of intramuscular bleeding and haematoma.^7^^,^^10^ In some anatomical areas this also presents further risk, such as the calf where bleeding might theoretically contribute to compartment syndrome, risking circulation to the foot.^7^ Such concerns might lead to patients being denied access to this therapeutic intervention.

While injection of BoNT in the management of spasticity may be performed using land-mark identification, accuracy of delivery into target muscles can be as low as 40%.^11^, ^12^, ^13^, ^14^, ^15^ Techniques such as electro-stimulation, electromyogram, and ultrasound image (UI) guidance can be used to improve localization accuracy and safety with the potential for improved outcomes.^11^, ^12^, ^13^, ^14^, ^15^ The use of UI to improve localization is an area of rapid clinical expansion due to the ability to directly visualize: (1) contractile tissue, including delineating different muscles within an anatomic compartment; and (2) injection risk areas, such as the neurovascular bundle and theoretically reduce the risk of an adverse event.^7^^,^^16^ Technological innovations also mean that access to portable, low-cost ultrasound imaging systems has further reduced barriers to using ultrasound.^7^^,^^10^^,^^16^^,^^17^

However, the use of an ultrasound modality by clinicians without a formal background in imaging raises questions, including: (1) what is the scope of the UI practice when performed by a clinician managing spasticity; (2) what level of UI education to achieve competency, is necessary for the safe and appropriate performance of an image guided procedure such as BoNT injection in spasticity management; and (3) what governance considerations are relevant for a clinician performing an image guided BoNT injection for spasticity management?

In considering the above questions, we are mindful that UI may have other uses in spasticity management, such as sonographic assessment of spastic or contracted muscle. However, using ultrasound in diagnostic assessment of muscle in the context of spasticity management is not common or routine practice and research is needed to identify parameters, processes, and utility of such an approach.

A framework approach has been previously used^16^^,^^18^, ^19^, ^20^ to define and align the elements of scope of practice (Scope), education for competency and governance for clinicians performing point of care ultrasound (PoCUS) imaging – as a mechanism for consolidating and expanding access to PoCUS. The framework approach (fig 1; explanation of terms in table 1) is premised upon the inter-relationship of each of the components, whereby explicit alignment of each of the components provides the foundation for consolidation and expansion of practice. This paper therefore shares some generic content with other areas of practice for PoCUS and we aim to provide a consistent approach to implementation and practice development support.^16^^,^^18^, ^19^, ^20^

**Fig 1:**
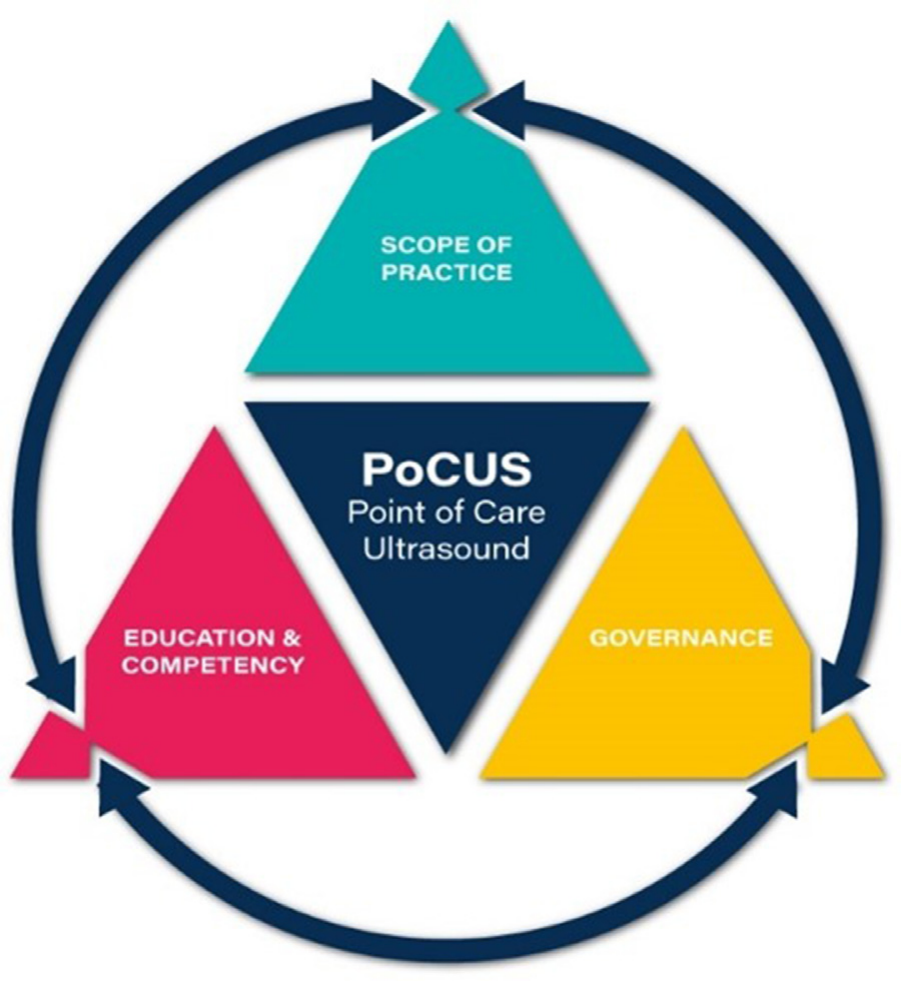
PoCUS framework triangle; adapted for UI in spasticity management (20). A framework for PoCUS. Concept by Dr Mike Smith (Cardiff University UK), created by Dan Molloy (freshwater.media). Copyright 2021 Dr Mike Smith

**Table 1:** Definitions of Scope, education for competency and governance

Term	Key elements	Additional information
Scope of practice (Scope)	Refers to the context and scope of the UI performed *plus* (any) interpretation / reporting of that UI *plus* (any) clinical decision making informed by that UI.	Scope allows for specifying any UI that is *not* going to be performed; and/or where UI is performed any interpretation / reporting *not* undertaken; and/or where UI is performed any clinical decision making *not* informed by the UI.
Education for competency	Refers to the UI education undertaken (both informally and formally) and subsequent assessments of competency.	Transparent, purposeful, and efficient UI education provision and competency assessments are made possible by aligning with the Scope. Appropriate UI education and competency are key contributors to safety and governance.
Governance	Includes legal and professional permissions (professional *and* regulatory body – if different), insurance arrangements and quality assurance	These are in part informed by the Scope; and by professional and local/national agreements; and via care pathway arrangements.

The objective of this work was therefore to establish scope of practice, competency (through education) and governance for ultrasound image guided injection of botulinum toxin in the management of spasticity using the framework approach. To establish these elements the opinions of an international, cross-disciplinary, purposively selected group of experts were convened using an online Delphi methodology. The principles in the ‘Recommendations for the Conducting and REporting of DElphi Studies (CREDES)’ were used in conducting and reporting this Delphi study (fig 2).^21^

**Fig 2:**
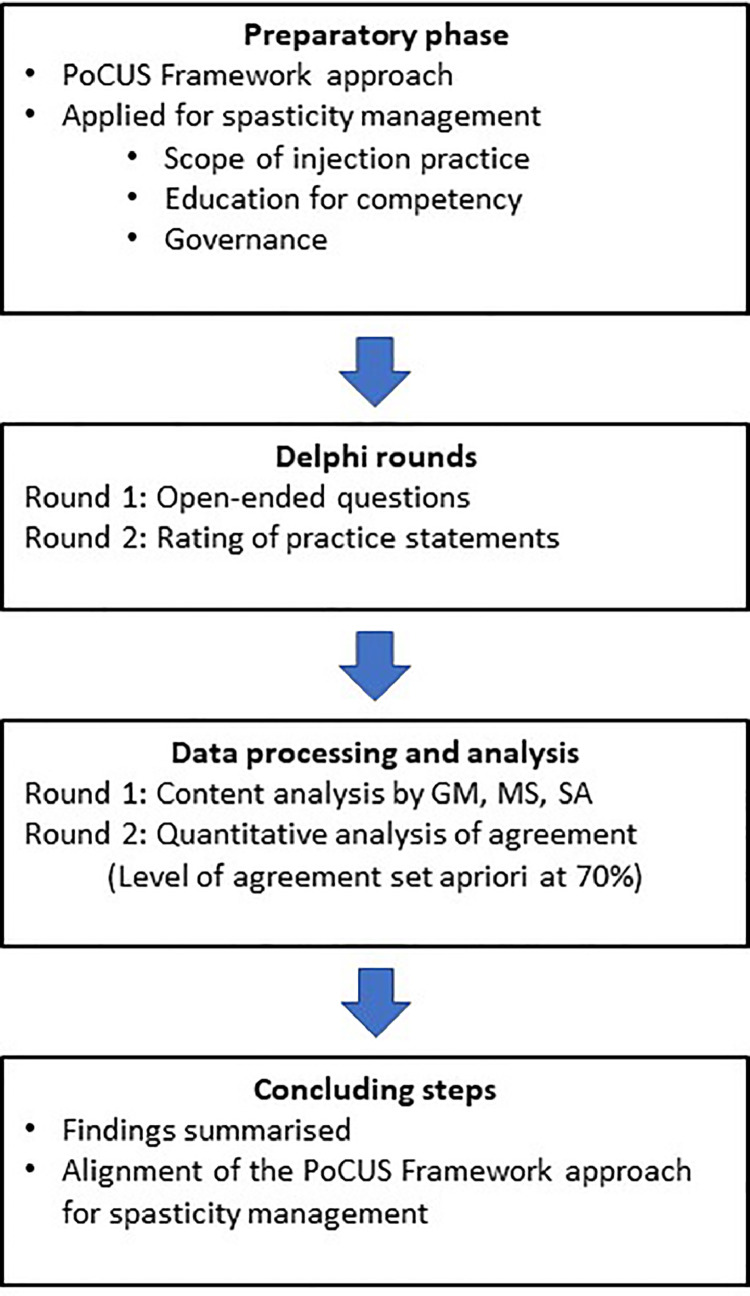
Conducting and Reporting of Delphi Studies (CREDES) study flow diagram.

## Methods

### Study design

An online Delphi methodology was used, comprising an initial open first round consultation to facilitate ‘idea generation’, with subsequent round(s) aiming to achieve consensus where possible.^22^^,^^23^ The option was available to undertake as many rounds of consultation as needed to achieve saturation in the views given. If consensus was not possible this was documented as a finding of the study.

### Participants

Purposive sampling was used to recruit ‘expert’ participants, defined as regularly using UI to administer BoNT for the management of spasticity and having contributed to research/guidance related to the management of spasticity. ‘Experts’ were identified via a combination of their involvement in spasticity management publications and/or conference presentations and/or national/international guideline involvement. Delphi sample sizes can vary significantly depending on the complexity of the objectives and heterogeneity of the participants.^22^^,^^23^ However, the population (‘experts’) was considered to be relatively homogenous and so in keeping with sample size recommendations for such a group, we aimed to recruit 15-20 participants.^24^

### Ethical considerations

Ethical approval was granted by the School of Healthcare Sciences, Research Governance and Ethics committee, Cardiff University. Invitations were disseminated with written information about the study and participants were given the opportunity to ask questions. Consent to participate was assumed from participants’ response to the invitation and subsequent completion of the Delphi survey.

### Consultation process

The Delphi was undertaken online using ‘Online surveys’ (https://www.onlinesurveys.ac.uk), a web-based survey and questionnaire platform. Data collection was conducted between October 2021 and March 2022.

#### Round 1

Open-ended questions were posed to generate participants views regarding: (1) scope – potential uses of UI in the management of spasticity, including the administration of BoNT or phenol. The application of US in reviewing the structure of muscle was also considered regarding possible changes seen when muscle has become more contracted over an extended time period; (2) education for competency; and (3) governance.

This included some pre-prepared statements developed from the existing literature.^25^ For competency and governance elements, participants in the Delphi study were asked for their opinions on UI competencies and governance relating to a specific set of circumstances: (1) pertaining solely to Scope 1 [administration of BoNT; see table 4]; and (2) where the clinician was already competent and experienced in the administration of botulinum toxin (eg, clinical reasoning, dose selection, reconstitution/dilution, manual localization and injection), but was UI naïve (ie, no previous experience or training in UI).

After round 1, 3 researchers (GM, MS, SA) independently completed content analysis of the qualitative round 1 data to minimize bias. All statements produced from the round 1 analysis were presented back to the panel in round 2.

#### Round 2

In this round, the panel were asked to rate their level of agreement with the statements developed from round 1 using a 5-point Likert scale ranging from 'strongly agree' to 'strongly disagree' along with the option for making free text comments.^26^^,^^27^

Quantitative analysis of responses was carried out with overall level of agreement for each statement calculated. Level of agreement (where participants ‘strongly agreed’ or ‘agreed’ with a statement) was set *a priori* at 70%,^23^^,^^24^ with statements meeting or exceeding this accepted; and rejected if they did not. After analysis of round 2 data, high levels of agreement were achieved (see results) by the panel; therefore, further Delphi rounds were not considered appropriate.^22^^,^^28^^,^^29^

## Results

### Participants

Fifteen participants were recruited to the ‘expert’ panel (table 2), all of whom completed rounds 1 and 2 of the survey.

**Table 2:** Demographics of Delphi participants

Characteristics	Number and proportion of respondents
Sex (n; %)	Male (9; 60%); Female (6; 40%)
Profession (n; %)	Physician (6, 40%), physiotherapist (8, 53%), occupational therapist (1, 7%)
Clinical setting for treating patients*	Outpatient (14, 93%), inpatient (12, 80%), in the community (4, 27%)
Geographical base†	Europe (13, 87%), North America (2, 13%).

⁎ Total exceeds n=15, 100% because some participants reported working in more than 1 setting.

† Majority of European based participants were from the 4 nations of the UK (England n=7, Wales n=2, Scotland n=1, Northern Ireland n=1); the other European participants were based in Austria (n=1) and Spain (n=1). Both North American participants were based in Canada.

#### Round 1 and 2

A summary of the content analysis undertaken after round 1 is shown in table 3; while the statements and accompanying levels of agreement from round 2 are shown in table 4. As the *a priori* threshold agreement level was achieved for all statements in round 2 (with no free text comments of note), further rounds of consultation were not deemed necessary.

**Table 3:** Content analysis undertaken after round 1

Section	Decisions taken with rationale
Scope of practice	Refinement of the wording for Scope statements 1 and 2 were made in response to participants’ comments. The third Scope statement was retained in its original form.
Competency	No major changes to the competencies were made, but modification to sentence structure for clarity was undertaken. An additional competency was added relating to image capture and storage.
Governance	Collapsing of the governance statements into a single statement for consideration was undertaken.

**Table 4:** Consensus statement agreement from Delphi round 2

Scope of practice statements	Level of agreement n (%)
1. Visualization and identification of target muscle and neighboring tissues (including neurovascular structures). Visualization of needle passage and subsequent localization of botulinum toxin into target muscle; avoiding at risk structures.	15 (100%)
2. Visualization and identification of target muscle or nerve; and neighboring tissues. Visualization of needle passage and subsequent localization of injectate such as phenol into target tissue; avoiding at risk structures.	12 (80%)
3. *Evaluation of muscle structure, thickness, and composition to aid clinical assessment and decision making.	12 (80%)*
**Competency statements**†	
A. Understand foundational physics as applied to UI, including how the UI is generated.	13 (87%)
B. Understand and demonstrate how ultrasound settings can be adapted to optimize imaging, including the management of artefacts (including anisotropy).	15 (100%)
C. Able to identify different tissue types and anatomic structures on UI.	15 (100%)
D. Able to apply injection specific ultrasound strategies including in-plane and out-of-plane localization and needle enhancement techniques.	15 (100%)
E. Understand thermal and non-thermal effects of ultrasound and precautions including ALARA (As Low As Reasonably Allowable) principle.	11 (73%)
F. Understand and demonstrate adherence with infection control procedures specific to UI guided invasive procedures.	15 (100%)
G. *Able to capture and store ultrasound images of localization into target muscle.	12 (80%)*
**Governance statement**†	
The practitioner should consider ultrasound imaging governance relevant to their country of practice and professional regulator.	15 (100%)

⁎ Denotes a statement where respondent(s) indicated some ‘disagreement’. Where other statements had less than 100% agreement, the non ‘agree/strongly agree’ respondents indicated they ‘neither agreed nor disagreed’.

† The competency and governance statements relate specifically to Scope statement 1.

## Discussion

The responses from the expert group led to 3 scopes of practice being generated, relating to the use of UI in the management of spasticity. Specifically relating to Scope 1 (ultrasound image guided injection of BoNT), a range of key competency requirements were agreed upon, alongside a singular, broad governance statement.

A key aim of this paper is to support the consolidation and expansion of UI in spasticity management, thereby optimizing access for patients to safe and effective UI use in spasticity management. Given the potential for permission and governance complexities around clinicians (who are unlikely to have a background in medical imaging) using UI, the PoCUS framework approach provides an integrated approach to supporting robust clinical practice.

### Scopes of practice

Scope 1 (table 5) provides clarity on a primary role for UI in performing guided injection of BoNT in the management of spasticity, supported by 100% agreement of the participants in the second Delphi round. The role of education/competency and governance considerations (fig 1 and table 1) to provide the foundation for sustainable practice will be considered later in this discussion section. Given the potential for UI guidance of BoNT to provide improvements in ease of treatment and patient safety (for some injections and possibly better clinical efficacy),^8^^,^^30^ this is an important study outcome in supporting such practice.

**Table 5:** Scope in the management of spasticity

Scope	Rationale for Scope	Excluded from Scope*
1. Visualization and identification of target muscle and neighboring tissues (including neurovascular structures). Visualization of needle passage and subsequent localization of botulinum toxin into target muscle; avoiding at risk structures.	Allows the clinician to (i) identify the tissue that is the therapeutic target and observe delivery of the injectate (*treatment efficacy*); and concurrently (ii) avoid neighboring and at-risk tissues (*reduced risk of iatrogenic harm*).	Sonographic assessment of target tissue† (e.g., muscle structure, thickness and composition). Sonographic assessment of other tissues in the field of view (e.g., thromboli, presence of space occupying lesions including intra-muscular lesions such as sarcomas, etc.) ‡
2. Visualization and identification of target muscle or nerve; and neighboring tissues. Visualization of needle passage and subsequent localization of injectate such as phenol into target tissue; avoiding at risk structures.	Allows the clinician to (i) identify the tissue that is the therapeutic target and observe delivery of the injectate (*treatment efficacy*); and concurrently (ii) avoid neighboring and at-risk tissues (*reduced risk of iatrogenic harm*).	Sonographic evaluation of target tissue† (eg, nerve root structure, thickness and composition). Sonographic assessment of other tissues in the field of view (e.g., thromboli, presence of space occupying lesions including intra-muscular lesions such as sarcomas, etc.) ‡
3. Evaluation of muscle structure, thickness, and composition to aid clinical assessment and decision making. §	Allows the clinician to combine sonographic findings with clinical assessment, as part of their reasoning process.	Sonographic assessment of other tissues in the field of view (eg, thromboli, presence of space occupying lesions including intra-muscular lesions such as sarcomas, etc.) ‡

⁎ Reflecting the PoCUS framework approach, explicit *exclusion* of other potential sonographic roles (and communication of these exclusions) provides clarity for a range of stakeholders (see table 6); and allows for expedited sonographic training.

† Note that where a clinician has undertaken appropriate training and can demonstrate competency in these elements, these roles can be undertaken in parallel to Scope 1 and Scope 2.

‡ Nonetheless, if a clinician has an elevated index of suspicion, they have responsibility to seek a second opinion and/or escalate;

§ Scope 3 would be a clinician (who was performing Scope 1 and/or Scope 2) but with a more advanced UI competency.

Scope 2 (table 5) reached the threshold for acceptance (actual agreement rate = 80%); though multiple expert participants reported in the round 1 free text comments that they did not perform phenol nerve blocks in their current practice. Scope 2 can therefore be considered an emerging spasticity management approach,^9^ perhaps reflecting the (actual or perceived) risk associated with nerve root blocks and ablation. If this is indeed the case, mechanisms to support robust use of UI guidance (to be discussed later in relation to UI guidance of BoNT) have the potential to address some barriers to such services being established.

Scope 3 (table 5) also reached the threshold for acceptance (actual agreement rate = 80%). However, several expert participants noted in the round 1 free text comments that this would require additional sonographic training, as this scope involves ‘sonographic assessment’ rather than simply ‘image guidance of a procedure’. This last point highlights a crucial delineation regarding the use of UI to solely perform an image guided BoNT (Scope 1), where thetephenn to perform the technique is a clinical one and with the role of UI solely to ensure safe and accurate delivery of BoNT.

By purposefully excluding other UI roles (column 3 in table 5; namely sonographic assessment and nerve ablation, which are areas emerging in practice), expedited training in UI guided BoNT can be achieved (see later section: education and competency). Such training can help reduce barriers to service expansion; and ensure that patients whose risk factors may preclude the use of non-image guided BoNT can more readily access these valuable spasticity treatment adjuncts. An additional benefit is that for health care systems and/or professional groups who are not currently permitted to use UI, Scope 1 provides a clinically meaningful but also tightly defined UI practice guide. Drawing upon the principles in this paper, it is hoped that this will provide leverage for such health care systems and professional groups to lobby for acceptance of performing such roles and for clinically appropriate development into practice in scope 2 and 3 in due course.

Aligning with the PoCUS framework approach, table 5 presents the 3 Scopes generated from this Delphi process as well as exclusions. Whilst identifying areas of UI practice not to be performed may seem counter-intuitive, this approach confers numerous advantages, as per table 6.

**Table 6:** Governance and care pathway benefits of describing scope of sonographic (and clinical) practice

‘Stakeholder’	Utility of defining the Scope
Other members of the care pathway	Other members of the care pathway are aware of what the clinician is undertaking the scan for; and what can be inferred from the scan. Just as importantly they are aware of the limitations of the scan and that for aspects that are out of scope of practice (eg, thromboli, presence of space occupying lesions including intra-muscular lesions such as sarcomas, etc.) that the scan is not for the purposes of either confirming or excluding.
Patient	In providing informed consent, the patient is aware of what the imaging is being performed for; but just as importantly what the imaging is not being performed for (as above).
Professional body and regulatory body; including insurer	The professional and regulatory bodies can see if the imaging being performed (and where appropriate, any clinical inferences derived from the scan) align with the scope of the profession. Depending upon the professional, regulatory and insurance environment, such information can inform whether there is professional permission to proceed, professional indemnity coverage and determine the level and cost of liability coverage.
The manager of the clinician	Provides clarity regarding what the clinician will be performing UI for and what they will be doing with that information. As such, allows for the design and staffing of existing and new care pathways.
The education provider	Provides clarity regarding the requisite education content and the necessary areas for evidencing competency. This will be in parallel to injection specific elements (e.g., aseptic technique, pharmacology, etc.).
The clinician	The clinician can undertake the necessary education and competency assessment requirements; and can be confident that the relevant governance elements have been addressed and that other members of the care pathway are aware of the remit of the scan.

### Education/competency

As per table 1, education and competency refer to both formal and informal training and assessments of competency in relation to injection of botulinum toxin. Noting the ‘Scope’ section (above; including table 5), a noteworthy advantage of the defined scope is that these purposefully do not require education and competency in ‘sonographic assessment’. This provides the opportunity for a highly focused, ‘UI guided injection’-specific training route.

Nonetheless, a range of UI skills can be considered essential so that high quality clinical practice can be assured. Reflecting this, competency statements A to G (in table 4) were generated from round 1 of the Delphi study and all surpassed the 70% agreement threshold in round 2.

UI competency statements B, C, D and F all attained 100% agreement and can be considered to reflect core aspects of ‘UI guided injection’-specific training and practice. Compared to these, UI competency statement A could be of lower relevance; however, we emphasize that it is a necessary component for an UI operator (including a clinician performing an UI guided injection) as part of understanding the considerations and limitations of UI.

The lower level of agreement for UI competency statement E may be explained by the limited scan time (and thus limited tissue exposure to ultrasound energy) of an UI guided BoNT injection; the low/non likelihood of imaging higher risk tissues (eg, fetal tissue, orbit region;^31^), contrasted with the greater perceived risk around use of cytotoxic injectates. Nonetheless we advocate its inclusion, as this forms part of the risk assessment of using UI.

The lower level of agreement for UI competency statement G may in part reflect the challenge of storing cine-loops within the limited capacity of existing systems such as PACS (Picture Archiving and Communication System). However, the ability to capture and retain footage in the electronic medical record (EMR) of the injectate being accurately delivered is a valuable medicolegal mechanism.

In terms of advocating a specific UI course structure for clinicians looking to undertake UI guided BoNT injections, this is beyond the remit of this paper. However, we encourage educators, clinicians and service providers to consider the following: (1) the undertaking of a formal course or equivalent training (e.g., through a Higher Education Institution or equivalent) where all the requisite UI components (relevant to the subsequent Scope to be undertaken, ie, table 4, A-G) are covered and formally assessed – should provide a robust foundation for high quality UI practice; and (2) the specifics of whether injection practice is learnt (and formal competency demonstrated) as a pre-cursor to the above UI training – or is integrated with the above UI training – is likely to be determined on a course-by-course basis.

### Governance

As per table 1, governance refers to a range of different aspects such as legal and professional permissions, insurance arrangements and quality assurance.

In Delphi round 1 (‘concept generating’), participants were asked about their opinions on whether (i) permission from their employer or other members of the care pathway and (ii) insurance provision or coverage were necessary; and any other governance considerations they felt were relevant to use of UI in this limited role (ie, Scope 1; UI guided injection of BoNT). Regardless of profession, clinical setting for treating patients or geographic base of the participants, participants’ responses were highly heterogenous, ranging from agreeing that one, both or neither of (i) and (ii) were necessary considerations when UI was used in this limited role. This may reflect complexities, variability and uncertainty around regulation, professional autonomy and insurance or indemnity arrangements in relation to the use of UI; as well as the nuances of different health care system (in different countries and jurisdictions) and care pathway arrangements.

Given the above heterogeneity, for Delphi round 2 (‘evaluating agreement’) the decision was made to condense the governance statements to “The practitioner should consider UI governance relevant to their country of practice and professional regulator”. This attained 100% agreement from respondents and illustrates that while there may be a range of circumstances (as noted by one participant: “*Governance considerations need to be unique to each region and country*”), expert consensus was that such aspects must be considered and addressed.

In terms of the UK (where the researchers; and many of the Delphi participants are based), UI is an unregulated modality, meaning there are no legal barriers to its use. In terms of individual professions in the UK: use of UI is not explicitly excluded from the practice of physicians; and for physiotherapists it is accepted as being within their professional scope of practice where UI is demonstrably used as part of physiotherapeutic assessment and/or treatment.^32^^,^^33^ Nonetheless, as with any other technique or modality, the clinician is required to be appropriately trained and be able to demonstrate competency.^32^^,^^33^ This mirrors the framework approach, regarding defining the Scope and aligning this with the subsequent education and formal competency assessments. In producing, National UK Point of Care Ultrasound (POCUS) guidance for physiotherapy practice, the framework using in this work, has been reviewed by the national professional body.

However, it is noted that in many health care systems, various professions (physician, allied health professional, nurse, etc.) may not be permitted to use UI. Drawing upon the principles outlined in this paper – and the precedent set by professionals in other health care settings such as the UK – we believe that expansion of service delivery models can and should occur for the benefit of patients.

## Study limitations

This Delphi study incorporated the views of health care professionals based in the UK and other countries with high income health care settings (Canada, Spain, Austria). This limits the external applicability of the Delphi findings and risks presenting views that are biased toward such settings. However, it is noted that such settings also have some of the most progressive models of UI, whereby physicians, allied health professionals and nurses are increasingly able to expand their scope of clinical practice to include advanced practice components (including pharmacologic interventions such as BoNT injection; and UI).

It is also noted that the Delphi participants were recruited based upon their expertise in BoNT as opposed to phenol or sonographic assessment of changes secondary to spasticity. This may explain the lower levels of agreement for Scope 2 and 3. However in this emerging area of clinical practice, UI guided injection of BoNT is arguably the most established role for UI in spasticity management. Nonetheless, we encourage the neuro-rehabilitation community to consider Scope 2 and Scope 3 as potential expansions, which are likely to enhance practice in this context.

## Conclusions

This paper has drawn upon the findings of an international, cross-profession Delphi study to inform the proposal of 3 Scopes relating to the use of UI in spasticity management, with particular emphasis placed on UI guided injection of BoNT. By adapting a framework approach for PoCUS, this paper also presents governance considerations and competency requirements in areas including image optimization and interpretation, needle visualization and safety. The framework approach can be used to facilitate the consolidation and expansion of access to UI guided injection of BoNT for spasticity.

Two further Scopes (UI guided injection of phenol; and sonographic assessment of the spastic/contracted tissue) are also described. Whilst acknowledging that some health care systems and professional groups may not be permitted to undertake the Scope(s) outlined in this paper, we advocate that the principles and precedent can be used to support progressive practice.
